# Life History Strategies of the Winter Annual Plant *Echinops gmelinii* (Asteraceae) in a Cold Desert Population

**DOI:** 10.3390/plants14020284

**Published:** 2025-01-20

**Authors:** Yanli Wang, Xinrong Li, Jiecai Zhao

**Affiliations:** 1 College of Forestry, Gansu Agricultural University, No. 1 Yingmen Village, Anning District, Lanzhou 730070, China; 2 Shapotou Desert Research and Experimental Station, Northwest Institute of Eco-Environment and Resources, Chinese Academy of Sciences, 320 Donggang West Road, Lanzhou 730000, China; lxinrong@lzb.ac.cn (X.L.); zhaojc@lzb.ac.cn (J.Z.)

**Keywords:** population dynamics, winter annual, seed germination, seedlings survival, soil seed bank, temperate desert

## Abstract

*Echinops gmelinii* Turcz. is a winter annual species of the Asteraceae family, distributed in sandy areas of northern China, and is crucial for wind avoidance and sand fixation. To understand the inter- and intra-annual population dynamics of *E. gmelinii* in its cold desert habitats, we conducted long- and short-term demographic studies to investigate the timing of germination, seedling survival, soil seed bank and seed longevity of natural populations on the fringe of the Tengger Desert. Cypselae (seeds) of *E. gmelinii* can germinate in both July and August, but this process is heavily affected by precipitation amount and timing. Early emerging seedlings died rapidly under the high temperature and drought stress, before completing their life cycle. Later emerging seedlings could survive to complete their life cycle due to more suitable conditions for plant growth. In short, seedling survival dynamics were affected by precipitation distribution, and the survival rates were low (<4%). In addition, we found that the high seed production (1328 seeds·m^−2^) of *E. gmelinii* depended mainly on the production of seeds by individuals rather than high plant density (35 individuals·m^−2^). The contribution of newly ripened seeds and soil seed banks to seedlings emergence was 57.7% and 42.3%, respectively. Thus, only a small amount of the newly matured seeds was depleted during the year. Only 23.6% of the annual seeds germinated, and the remainder accumulated in a persistent soil seed bank (seed longevity was ≥2 y). The amount and timing of precipitation distribution were the key factors affecting the population dynamics of *E. gmelinii* in our study area. This species can cope with the uncertain precipitation patterns though a “cautious” germination strategy, varying the timing of germination and forming a persistent soil seed bank.

## 1. Introduction

Increased drought frequency and intensity are among the most serious impacts of current and future climate change. Life history strategies of plants in response to environmental variation are an important theme in evolutionary ecology [1]. Seed bank size, seed germination and seedling survival help determine the adaptive capacity of a species to varied environments, enabling us to interpret and forecast ecological dynamics, especially for therophytes in desert areas [2,3,4]. Much research has shown that therophytes exhibit two disparate germination mechanisms, including rapid seed germination and early seedling settlement. Thus competitive strength of the plants can be improved, with seed germination delayed, which is conducive to spreading the risk for plants in an unpredictable environment [5]. It is well understood that seed germination has been affected by various variables in laboratory experiments, including temperature, moisture and light [6,7,8]. However, seedling survival, seed germination and seed bank dynamics are infrequently studied via long-term field monitoring [9].

In the desert habitats, seeds need adequate water conditions to germinate, and consistency in wet circumstances may increase seedling emergence [10,11,12,13]. Drought stress was the primary cause of seedling death, and seedling survival showed a significant positive correlation with the water content in the 0–20 cm soil depth [14]. Apart from seedling survival, soil moisture helps subsequent phases of the life cycle by promoting growth, reproduction and seed accumulation [15,16,17,18]. Ephemeral plants germinate after spring precipitation and complete their life cycle before the high temperatures and drought stress of summer [19]. Especially for winter annuals distributed in temperate sandy regions with sparse rainfall and uneven seasonal distribution, seedlings experience a long period of drought, as well as high-temperature and low-temperature stress after seed germination in summer or autumn [20]. Therefore, the winter annuals distributed in this region must have special adaptive strategies. So far, there are few studies on survival strategies of winter annuals population in temperate desert regions.

Seed banks are essential for the sustained development of populations in arid ecosystems, as we cannot forecast the prospects for seedling establishment or seed germination [21,22]. Germination in soil seed banks is likely to be delayed until conditions are suitable for seedling establishment [23]. Especially for therophytes, seed banks are critical to the development and genetic diversity of populations [24,25], so the only way to survive in dangerous and unpredictable environments is by forming a seed bank [26]. Hence, understanding the characteristics that affect seed banks’ composition and dynamics would provide insight into the future responses of populations and ecosystem functions to climate change [27].

Being one of the most frequent winter annuals in northern China’s temperate desert, *Echinops gmelinii* Turcz. is also a dominant pioneer herb after sand stabilization. Our previous studies found that the life process of *E. gmelinii* is highly influenced by changes in precipitation patterns [28]. Thus, an important future question is whether soil seed banks can sustain population persistence through unpredictable precipitation and multiple years of low reproduction. In addition, the mechanisms by which life history strategies for seed dynamics and seedling survival are adaptations of winter annuals to temperate desert habitats are poorly understood [29]. Thus, we hypothesized that soil seed banks could sustain the population persistence under unpredictable precipitation patterns. To test this hypothesis, controlled experiments and long- and short-term demographic field observation were conducted to (1) explore the inter-annual patterns of seedling survival and seed germination; (2) quantify the contribution of freshly mature seeds or soil seed bank seeds to seedling emergence; and (3) determine the effects of seed longevity on seed germination and viability. In summary, at the regional scale, we explored the adaptation strategies of winter annuals to the unpredictable cold desert environment that result in sustainable population development. The findings could offer a theoretical foundation for comprehending plant population growth response to climate changes in cold deserts.

## 2. Materials and Methods

### 2.1. Research Site

We conducted the research at the Shapotou Desert Research and Experimental Station, located on the southeastern edge of the Tengger Desert (37°32′ N, 105°02′ E). Our research site is in a temperate (cold) desert with a continental climate. The average temperature is 9.6 °C, with −6.9 °C in January and 24.3 °C in July. The absolute lowest temperature is −25.1 °C, while the absolute highest temperature is 38.1 °C. The temperature difference in a year is 63.2 °C, and the accumulated temperature ≥ 10 °C is 3017 °C. The first frost period appears in late September, while the last one is in mid-April. The growth period of plants is up to 150~180 days. Within the recent 60 years (1955–2015), the average precipitation in a year is 186.6 mm, and precipitation varies greatly at different times of the year. The annual distribution of precipitation is affected by the monsoon, and the seasonal distribution of precipitation is very different. The precipitation is mainly in the period from June to August (summer), i.e., 57.2% of the total precipitation for the year, whereas from December to February (winter) it is only 3.9%. Precipitation from April to October in the growing season accounts for 91.4% of the annual precipitation. Due to its location in the arid desert area, the potential annual evaporation is large, which is 2900 mm. The average annual wind speed in the area is 2.9 m·s^−1^. All precipitation data were collected from the weather station at the Shapotou Desert Research and Experimental Station. There is a mean number of 272 sand-driving wind events, with a wind speed of 5 m·s^−1^ per year, mainly from March to May.

### 2.2. Experimental Design

#### 2.2.1. Establishment of Plots for Studying Seedling Emergence and Survival Dynamics

Seedling emergence and survival of *E. gmelinii* were studied by randomly selecting sixty-five plots (50 × 50 cm) that were established between shrubs in March 2016 in restored vegetation districts covering 3000 m^2^, revegetated with *Artemisia ordosica* Krasch., *Caragana korshinskii* Kom., and *Hedysarum scoparium* Fisch., which had been planted in April 2013. The dispersal and germination unit of *E. gmelinii* is a kind of fruit called a cypsela, but it will hereafter be referred to as a seed. Seed germination time, seedlings emergence and survival through the life cycle were recorded continuously from March 2016 to October 2019. In the study area, the seed germination of *E. gmelinii* occurred from July to September. From 2016 to 2019, the number of newly emerging seedlings was recorded every day after precipitation until no further seedlings appeared. From July to October (summer and autumn) and from March to June (spring and summer) of the following year, seedling survival was recorded every 7–10 days until the completion of the life cycle. The criterion of plant survival was that it produced at least one seed. Seedling survival was not recorded from November to February (winter) due to low biological activity when the temperature is around or below zero in the study area.

#### 2.2.2. Establishment of Plots for Studying Seed Bank Dynamics

To investigate the characteristics of *E. gmelinii* seed bank dynamics, we carried out field survey experiments in a restored vegetation area covering 1925 m^2^ (35 cm × 55 cm) revegetated with *A. ordosica* and *C. korshinskii* and established in 2007 by Shapotou Station. Live plant density of *E. gmelinii* was recorded by randomly selecting 30 plots (50 × 50 cm) on 10 June 2017.

#### 2.2.3. Monitoring the Dynamics of Soil Seed Bank Soil Seed Bank Dynamics

In June and July 2017, 20 seed traps were randomly set in the study area to monitor the mature seed dispersal time and density of *E. gmelinii*. Plastic pots with holes at the bottom (height: 20 cm, diameter: 26 cm) were covered with non-woven cloth to form seed traps. The pots were buried in the soil and kept level with the ground surface, which was conducive to capturing *E. gmelinii* seeds. The seeds began to disperse by 15 June, and the seeds in the traps were collected every 15 d. The number of intact seeds (cypselae) and phyllaries without achenes was recorded. Then, the number of seeds per square meter was used to calculate seed density. This monitoring ended on 30 July 2017 after all seeds were dispersed.

For the purpose of investigating new mature seeds and soil seed banks’ contribution to seedling emergence, two 60 m transect belts were set diagonally within the study area in May 2017 (before the new mature seeds were dispersed), and a small plot of 50 cm × 50 cm was set at 10 m intervals on each transect. Twelve plots (50 cm × 50 cm) were covered with a nylon mesh (0.4 mm aperture) and represented the remnant soil seed bank, namely, the “covered plots”. In addition, another 12 “open plots”, without nylon mesh, were set adjacent to these plots, including newly matured seeds. These two groups of plots were used to investigate seedling emergence between July and September 2017. The seedlings in the “open plots” were from newly mature seeds and the remnant soil seed bank. In the “covered plots”, the nylon mesh mulch prevented the newly mature seeds from entering the soil, so the seedlings were only from the remnant soil seed bank. The contribution of the soil seed bank to seedling emergence was calculated as (seedling number from covered plots)/(seedling number from open plots) · 100%.

#### 2.2.4. Determining Seed Longevity

To determine seed longevity in natural habitats, newly matured seeds were collected in the study area in 2016. We put seeds in nylon mesh bags in the natural environment. To avoid germination when the seeds touch the ground, the mesh bags containing seeds had been placed on a wooden shelf 0.2 m higher than the ground. The seeds stored for 1 month were tested for germination. Seed viability and germination were tested after storage for 1 and 2 years on the wooden shelf in the field. We placed *E. gmelinii* seeds in 9 cm diameter glass Petri dishes and tested their germination in incubators under conditions of 12 h light/12 h dark 30/20 °C (optimum conditions). We added water as needed to maintain filter paper moist in the process of the test. We discarded the seeds that germinated, defined as the protrusion of the radicle (~2 mm long). We tested the non-germinated seeds for viability with 2,3,5-triphenyl tetrazolium chloride (TTC). We cut open and placed seeds in a solution of 0.5% aqueous TTC at 25 °C for 3 h. Embryos stained read or pink were considered viable, and those without stain as non-viable [29].

### 2.3. Statistical Analysis

To evaluate seedling survival dynamics over time, we analyzed survivorship curves by adjusting a log-linear model [30]. For the seed bank dynamics experiments, the number of seeds collected in seed traps and the number of seedlings in open (or covered) plots were converted to the number of seeds and seedlings per square meter (m^2^), i.e., seed yield and seedling density. We used a one-way ANOVA to analyze differences in final germination after seed storage for different periods of time in natural conditions and the seedling densities of *E. gmelinii* in open and cover plots. The averages were compared by Tukey’s HSD test, at a 5% probability level. To satisfy the conditions of ANOVA, the data were either arcsine transformed (i.e., seed germination) or logarithmic transformed (i.e., seedling density) before analysis. With the exception of seedling survivorship curves: the non-transformed data are displayed in all graphs. We performed all statistical analyses and figure creation using SPSS 18.0 (SPSS Inc., Chicago, IL, USA) and Origin 9.0, respectively.

## 3. Results

### 3.1. Seedling Emergence and Survival

**Figure 1:**
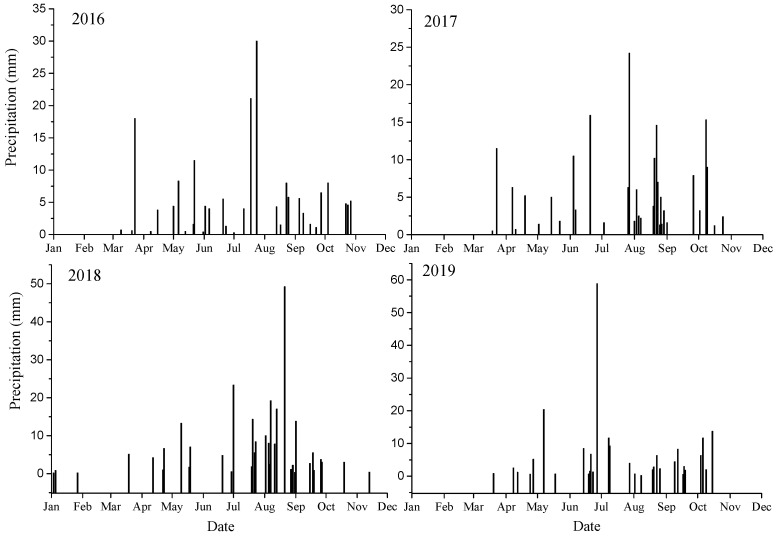
Daily precipitation in study area (Shapotou region) from 2016 to 2019.

During the experimental period, 95.3% (88.4% to 99.6%) of the total precipitation fell from April to October (growing season) on the study site, and 132 precipitation events were recorded, in which precipitation events <5 mm accounted for 59.1% and 20.5% of the total precipitation amounts. Additionally, 5–10 mm and 10–20 mm precipitation events accounted for 22.0% and 12.6% of the total precipitation events, respectively. Precipitation events >20 mm accounted for only 5.3% of the total events and 27.5% of the total precipitation amounts. Moreover, the largest precipitation events from 2016 to 2019 were 30 mm, 24.2 mm, 49.2 mm and 58.8 mm, respectively. In particular, the >20 mm precipitation events occurred in July and August from 2016 to 2018, while in 2019 they occurred in May and June (Figure 1).

In the study area, the seedling emergence of *E. gmelinii* was investigated for three consecutive years (from 2016 to 2018). *E. gmelinii* had two seedling emergence periods in summer. The first seedling emergence occurred after a large precipitation event in late July 2016, 2017 and 2018, with precipitation of 30 mm, 30.5 mm and 28.2 mm, respectively. The second seedling emergence occurred after 13.8 mm, 46.7 mm and 24.8 mm precipitation events in late August, respectively. In conclusion, the temporal variation of precipitation size and distribution is an important factor affecting seedling emergence time, and seedling emergence time is correlated with seasonal variation of precipitation. Furthermore, the number of seedlings varies greatly among different years, with the total seedling emergence from 2016 to 2018 being 908, 3578 and 329, respectively. The seedlings mainly appeared in the second seedling emergence period, and the first cohorts of seedling emergence accounted for 13.4%, 15.6% and 27.1% of the total seedling emergence, respectively (Table 1).

**Figure 2:**
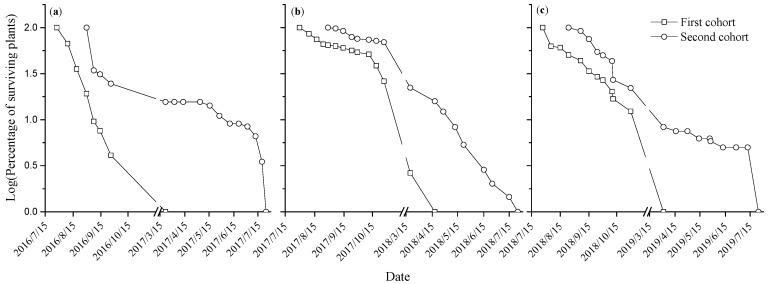
The characteristics of seedling survivorship curves of *E. gmelinii* during the life cycle of 2016–2017 (**a**), 2017–2018 (**b**) and 2018–2019 (**c**).

We monitored the seedling survival of *E. gmelinii* and found that seedling survival dynamics varied from year to year (Figure 2). In 2016–2017 and 2018–2019, the survival of the seedlings decreased rapidly at the early stage of growth and remained relatively stable after overwintering, while the initial survival of the 2017 and 2018 seedlings was higher than that of the 2016 seedlings. On the other hand, survival type differed between early and late cohorts. For the first cohorts of seedlings, only 4%, 26.2% and 12.4% of seedlings survived until the end of October 2016, 2017 and 2018, and few or no seedlings successfully overwintered and completed life cycle. The survival and fecundity of the second cohort, which appeared in late summer and early autumn were higher. Overall, the survival of the *E. gmelinii* population was low: only 3.1%, 1.2% and 3.65% of the 2016, 2017 and 2018 seedlings could complete their life cycle during the study period, respectively (Table 2). Thus, the *E. gmelinii* population was characterized by high emergence and low survival.

### 3.2. Soil Seed Bank Dynamics

During the seed dispersal monitoring period (15 June to 30 July 2017), 97.4% of seeds were dispersed between 15 June and 15 July, with 65% being dispersed in early July. In the study area, the live plant density was 35.7 ± 18.3 plants·m^−2^. The intact seed density was 1328.38 ± 230.25 seeds·m^−2^, and thus 75.2% of the inflorescences developed seeds (Table 2). Such outcomes implied that individual plants of *E. gmelinii* had high fecundity.

**Figure 3:**
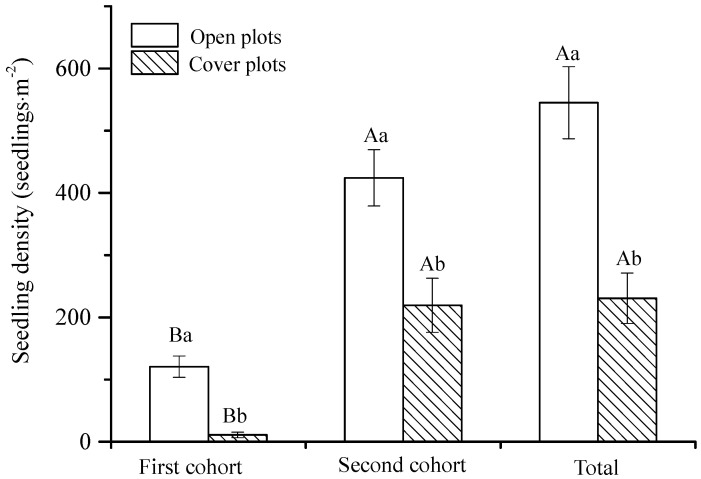
Characteristics of *E. gmelinii* seedling density in open and covered fields plots. Different uppercase letters on the bar chart indicate significant differences between the first and the second cohorts in each plot type (*p* < 0.05), and different lowercase letters indicate significant differences between the open and covered plots in each cohort (*p* < 0.05).

From July to September 2017, seedlings emerged in both open quadrats and covered quadrats with soil seed banks during the two emergence processes (Figure 3). Only a few seedlings emerged in the early stage (30 July, the first cohort), and the densities of open and covered quadrats seedlings were 120.67 ± 17.08 and 11.11 ± 4.53 individuals·m^−2^, respectively. A large number of seedlings appeared in the later stage (26 August, the second cohort), and the seedling densities in the open and covered plots were 424.33 ± 45.17 and 219.44 ± 43.36 individuals·m^−2^, respectively. Therefore, 57.7% of the total number of seedlings emerged from newly mature seeds, and the soil seed bank contribution to seedling emergence was 42.3%. Thus, the seeds in the soil seed bank contributed greatly to the population regeneration. According to the dispersal density of the mature seeds, 23.6% of the newly mature seeds germinated in the same year (Table 2, Figure 3).

### 3.3. Seed Longevity

**Figure 4:**
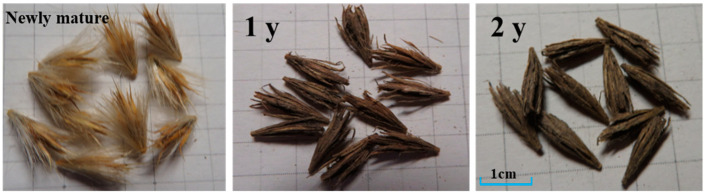
The morphological characteristics of the seeds after storage for 1 and 2 years under natural conditions.

**Figure 5:**
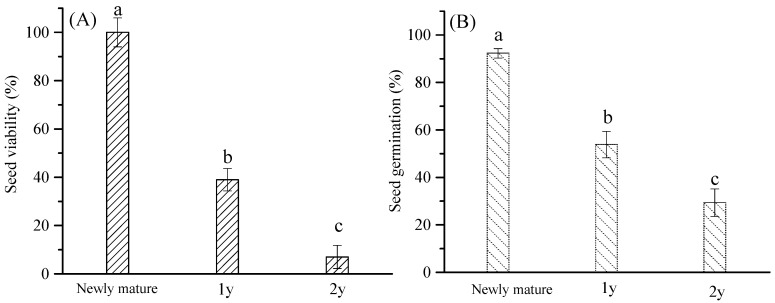
The characteristics of seed viability (**A**) and the germination (**B**) of *E. gmelinii* after storage for 1 and 2 years under natural conditions. Different lowercase letters on the bar chart indicate significant difference in seed viability and seed germination after different storage times (*p* < 0.05).

The seeds of *E. gmelinii* were surrounded by phyllaries and basal hair, which were dispersed by air from mature plants (Figure 4). The germination of newly mature seeds (storage for 1 month) reached 92.5%. After storage for 1 y, the germination was 53.7%, and 39% of the seeds were viable. The seed phyllaries were almost intact, but the outer basal hairs disappeared. After 2 y of storage, some of the phyllaries had decomposed, and most of the seeds were necrotic or rotten. Only 7% of the seeds remained viable, while the germination of the viable seeds was 29.1%. The results showed that seed longevity was at least 2 y in the natural habitats. In addition, seed germination decreased, and the seeds remained dormant, which was beneficial to the formation of the soil seed bank (Figure 5).

## 4. Discussion

In the Tengger Desert, *E. gmelinii* is the most frequent winter annual species, forming a persistent population and playing a vital role in maintaining sand fixation. However, information on the life history strategies of the winter annual responses to environmental stochasticity in a temperate desert is scarce. The traits of the soil seed bank and seed longevity relevant to population dynamics over time were identified by combining long-term observations of seed germination with the seedling survival demography of *E. gmelinii* and short-term observation and experimentation. Our results showed that the life cycle of *E. gmelinii* was characterized by high fecundity, seedling emergence and low survival percentages in the natural population. The seed germination of *E. gmelinii* occurred after a large precipitation event in summer or autumn. The seedlings that emerged at a later stage were more likely to survive and complete the life cycle than those that emerged early. Furthermore, the timing of seed germination and seedling survival is highly dependent upon the amount and timing of precipitation. In summary, to maintain population continuity, *E. gmelinii* has adaptive strategies such as cautious germination, flexible germination time and the formation of a persistent soil seed bank.

### 4.1. Inter-Annual Dynamic Changes of Seedling Survival and Seed Germination

Varied seed germination timing can reduce the mortality risk of seedlings, which is an effective strategy for plants growing in variable environments [31]. We found that there were two germination periods of the *E. gmelinii* population in the natural habitats, i.e., summer and autumn. The two periods of seedling emergence occurred in July and August from 2016 to 2018. Therefore, the variability of germination time and the characteristics of cohort emergence of *E. gmelinii* can share environmental variation risks and reduce seedling death risks, which is an adaptive strategy for plants growing in unpredictable environments. That is, the delay or advance of seedling emergence time may be the adaptive mechanism to the variation of precipitation patterns in the desert area. Some studies found seed germination has a close relation with precipitation and can only occur when precipitation reaches a certain amount [32]. The seedling emergence of *E. gmelinii* generally occurred after a precipitation event greater than 20 mm from 2016 to 2018 (Table 1). Thus, the seed germination of *E. gmelinii* might only occur after a heavy precipitation event, which belongs to the “cautious” germination mechanism. In addition, we found that seed germination could be caused by precipitation events of >10 mm in September and October 2019. This is due to increased seed germination after seed dispersal through multiple wet/dry cycles caused by precipitation, which enables rapid germination under transient soil moisture conditions [20]. This also suggests that precipitation amount required to induce seed germination may vary depending on seed germination mechanisms.

In semi-arid or arid areas, the number of seeds germinating has been closely related to the amount of precipitation. For example, seeds of the ephemeral plants *Lappula Redowskii* Hornem., *Pectis Angustifolia* Torr. and *Lepidium lasiocarpum* Nutt. growing in the northern Chihuahuan Desert of U.S.A. germinate after a rainfall event of >10 mm, while seed germination increases with precipitation in the range of 15 to 45 mm [33]. Beatley found that winter annuals need 10 to 15 mm of precipitation to induce germination, while a higher germination ratio requires more precipitation [34]. Similarly, in the cold Junggar Desert of northwest China, precipitation variability can create opportunities in some years for ephemeral *Diptychocarpus strictus* to germinate in the autumn and spring [35]. In our study, we found that a large number of *E. gmelinii* seedlings emerged after a continuous precipitation event with a total precipitation of 46.5 mm from 19 to 29 August 2017 (Figure 1, Table 1). A large number of seeds germinating after an extreme heavy rainfall event may lead to the depletion of the soil seed bank, which is not conducive to maintaining populations. In the desert areas, therophytes employ a germination strategy in batches, avoiding the emergence of all the seedlings after a heavy rainfall and the subsequent death in the drought. In addition, this may be the result of natural selection for long-term adaptive capacity to unpredictable harsh habitats.

The survival and growth of seedlings to maturity are critical for the persistence of plant populations in a given habitat. Since seedlings are very sensitive to changes in environmental factors; and the early growth of seedlings is easily affected by water availability winter annuals usually germinate in autumn, overwinter as rosettes and end their lifecycle before the summer drought [19]. The winter therophyte *E. gmelinii* has a long seedling period, with seedling survival being easily affected by environmental stress. In our study, a large number of *E. gmelinii* seedlings died after experiencing the drought and high-temperature stress in summer and low-temperature stress in winter, and only less than 4% of the seedlings survived to complete their life cycle (Table 1). In particular, the first cohort of seedlings all died after overwintering, and only the second cohort of seedlings survived to complete their life cycle (Table 1 and Figure 2). Thus, the seed germination time had an important effect on seedling survival. Moreover, we found that the seedling survivorship curves pattern of *E. gmelinii* was closely related to precipitation distribution (Figure 1 and Figure 2). The total precipitation from August to October in 2016 was only 60.3 mm, leading to the death of numerous seedlings due to drought stress. In particular, only 34.3% of the seedlings in the second group survived one week after emergence (Figure 1 and Figure 2a). Moreover, total precipitation in the period from August to October 2017 was 99.6 mm, so the soil moisture was sufficient, resulting in a high survival of seedlings after emergence, with a survival of 70% for the second cohort of seedlings at the end of October (Figure 1 and Figure 2b). Similarly, the total precipitation from March to June in 2017 and 2018 was 62.1 mm and 44.2 mm, respectively, so the survival of seedlings after overwintering showed a trend of rapid decline under the condition of low precipitation. Specially, the total amount of precipitation in the life cycle of 2018–2019 was 259 mm, and its survival and survival curve pattern differed little from that of the life cycle of 2016–2017. It may be due to the two extreme heavy precipitation events on 21 August 2018 and 17 June 2019, with precipitation of 49.2 mm and 58.8 mm, respectively (Figure 1 and Figure 2c). Therefore, the survival dynamics of seedlings are not only related to the amount of precipitation but also to precipitation distribution pattern. As shown in Figure 2a,c, *E. gmelinii* seedlings exhibited higher mortality during juvenile stages, implying mortality type III. While the seedlings survivorship curve in the Figure 2b displayed higher survival in the first stage, which suggested a type I survivorship curve [30]. In conclusion, the survival dynamics of *E. gmelinii* population were the result of the combined effect of seed germination time and precipitation distribution.

### 4.2. Dynamics of Seed Banks

Seed dispersal plays an important role in determining the temporal and spatial distribution of seeds, which affects the spatial type and dynamics of plant species at the population level [14]. As shown in Figure 4, *E. gmelinii* produces only one seed per ovary, which is surrounded by bracts and has white seta at the base [36]. Therefore, the long-distance dispersal of the seeds can not only increase the chance of random dispersal, but also avoid the competition of sibling offspring in the ecological niche. In our study, the seeds of *E. gmelinii* were scattered from 15 June to 30 July, and the seed density was as high as 1328 seeds·m^−2^, indicating that the plants had high fertility. In addition, 97.4% of seeds were dispersed between 15 June and 15 July, and 67.0% were concentrated in early July (Table 2). Our previous study found that the seeds of *E. gmelinii* had a certain degree of non-deep physiological dormancy after dispersal, and the dormancy state was broken after post-ripening [20,36]. Thus, gradual seed dispersal and non-deep physiological dormancy could disperse seed germination over time and thus reduce the risk of total germination, which might serve as an adaptive response of plants to changing environment [37].

Seed banks might be particularly critical for the persistence of therophytes, which possibly exist in the seed bank after the above-ground portion dies, awaiting favorable conditions to renew again [38]. The results of field investigation showed that seed germination occurred in the soil seed bank of *E. gmelinii* during two seedling emergence periods, contributing 42.3% to seedling regeneration. Also the germination of newly mature seeds accounted for 57.7% of total seedling emergence (Figure 3 and Table 2). Only 23.6% of the seeds that matured in the current year could germinate, indicating that most of newly mature seeds were in dormancy and formed soil seed banks. These seeds contained in banks are not only the foundation of the settlement, survival, reproduction and diffusion of plant populations, but also essential component of possible regeneration capacity of vegetation [39]. Especially in disturbed habitats, seed banks allow plants in seed form to endure several consecutive years without rainfall until environments are suitable for germination and emergence [40]. Darkness, alternate wetting and drying, sand burial and other conditions can cause *E. gmelinii* seeds to enter dormancy [20], which is good for soil seed banks’ formation. Furthermore, we found that 39.0% and 7.0% of *E. gmelinii* seeds were still viable after storage for 1 y and 2 y in the natural habitats, while the germination of viable seeds decreased and remained dormant (Figure 4). Therefore, we could conclude that seed longevity was at least 2 y, and the seed bank type is a persistent seed bank [29].

To sum up, rainfall patterns are changing under the background of changing climate, with increased extreme rainfall events, as well as rainfall intervals [41]. Climate change is increasing drought events’ severity and recurrence, possibly endangering various species [42]. We found that seed germination of *E. gmelinii* happened after a large precipitation event. Then, when there were more and more extreme heavy rainfall events, seeds mostly germinated within a year, resulting in soil seed bank depletion. In addition, severe drought caused by the increased precipitation interval was not conducive to the seedlings survival of *E. gmelinii*. Actually, only a few seeds germinated after heavy precipitation events in early (July), which might be due to the non-deep physiological dormancy of some newly matured seeds [20,36]. Also, most seedlings that appeared in late August or early September had a high survival (Table 1). Moreover, most of the newly matured seeds would enter and accumulate in the soil seed bank (Table 2, Figure 3), and the dark environment (sand burial), as well as hydration and dehydration cycles, could induce seed dormancy [28], leading to persistent soil seed bank formation. Therefore, the winter therophyte *E. gmelinii* has adaptive strategies, such as cautious germination, flexible germination time and formation of a persistent soil seed bank. In fact, although *E. gmelinii* has developed mechanisms for adapting to harsh environment of desert habitats, global changes are threatening population development, especially extreme precipitation events.

## 5. Conclusions

In the Tengger Desert, precipitation is scarce and seasonally uneven, with a high degree of unpredictability. In this harsh habitat, seedling survival, together with seed germination, is essential for plants’ life history, being of great significance for maintaining population development. Our research results have found that the life history of *E. gmelinii* is characterized by a high reproductive capacity and low seedling survival. In the natural habitat, seed germination depends on the amount and timing of precipitation, i.e., seeds have “cautious” germination. The early (late July to early August) emerging seedlings died rapidly under the high temperatures and drought stress, and all died before completing their life cycle. Later emerging (late August to early September) seedlings might survive to complete their life cycle. Seedling survival dynamics were affected by precipitation distribution, and survival was low (<4%). Both newly matured and soil seed bank seeds facilitate seedling recruitment. Moreover, the majority of freshly matured seeds can form persistent soil seed banks (seed longevity was ≥2 y). Thus, the timing of precipitation was the principal element influencing the population dynamics of *E. gmelinii* in our study area. This species has adaptive strategies such as cautious germination, flexible germination time and the formation of a persistent soil seed bank.

## Figures and Tables

**Table 1:** Seedling emergence and survival of *E. gmelinii* from 2016 to 2019 at the study site.

Process of Life Cycle	Date of Emergence	Single Precipitation of Inducing Seedling Emergence (mm)	Emerged Seedlings	Total Seedlings	Survived to Produce Seeds	Total Survival Ratio (%)
July 2016–July 2017	28 July 2016	30	122	908	28	3.08%
	28 July 2016	13.8	786			
July 2017–July 2018	30 July 2017	30.5	607	3578	43	1.20%
	26 August 2017	46.7	2971			
July 2018–July 2019	27 July 2018	28.2	89	329	12	3.65%
	24 August 2018	49.2	240			

**Table 2:** Seed release dynamics of *E. gmelinii* were observed from June to July 2017.

Density (Seeds·m^−2^)	Date of Statistics			
	30 June 2017	15 July 2017	30 July 2017	Total
Intact seeds	423.21 ± 72.95	871.20 ± 455.9	33.97 ± 6.38	1328.38 ± 230.25
Phyllaries without achenes	16.28 ± 11.6	404.81 ± 75.59	17.69 ± 3.83	438.78 ± 75.77

Note: the values in the table represent the mean ± SE.

## Data Availability

All data supporting the findings of this study are available within the paper. Should any raw data files be needed in another format, they are available from the corresponding author upon reasonable request.
